# A Case of Neoadjuvant Chemotherapy for Locally Advanced Inflammatory Breast Cancer With Low‐Level ER Expression

**DOI:** 10.1155/carm/6681740

**Published:** 2026-05-07

**Authors:** Qinwen Qin, Wenjun Jia

**Affiliations:** ^1^ Department of General Surgery, The Second Affiliated Hospital of Anhui Medical University, Hefei, 230601, Anhui, China, ahmu.edu.cn

**Keywords:** case report, inflammatory breast cancer, neoadjuvant chemotherapy

## Abstract

Inflammatory breast cancer (IBC) is a rare presentation of breast carcinoma characterized by tumor cell emboli invading the dermal lymphatics and manifesting as skin edema and redness. In this study, we report a case of locally advanced IBC with neoadjuvant chemotherapy. After the diagnosis of locally advanced IBC, this patient received a series of neoadjuvant chemotherapy treatment through the multidisciplinary team discussion, and thereafter, a modified radical mastectomy for left breast cancer and a split‐thickness skin graft were subsequently completed for this patient. Postoperative pathology revealed she achieved a pathological complete response (pCR). Following the recommendations of a further multidisciplinary team discussion, the patient continued on radiotherapy, a short course of endocrine therapy, and intensive therapy with capecitabine. During the 2‐year follow‐up period following the operation, the patient showed no signs of relapse or progression. The coordination of different disciplines is of paramount importance in ensuring the optimal treatment of locally advanced IBC patients, thereby facilitating a more favorable prognosis.

## 1. Introduction

Inflammatory breast cancer (IBC) is a rare and aggressive form of carcinoma, accounting for approximately 1%–5% of all breast cancers [1]. The typical presentation of IBC is characterized by the rapid onset of unilateral breast erythema, edema, and peau d’orange [2]. Early recognition and diagnosis of IBC is critical, as it is often initially misdiagnosed due to the similarity of the initial clinical symptoms to those of acute mastitis or abscess, resulting in treatment delays [3]. The young woman in this case report is confronted with the challenge of distinguishing diagnoses of mastitis, nonlactating mastitis, and nonspecific types of breast cancer. The treatment of IBC involves trimodal therapy including chemotherapy, surgery, and radiation therapy [4]. Although the prognosis for IBC is typically less favorable than that for noninflammatory forms, the completion of trimodal treatment is associated with improved locoregional control rates [5]. This finding reinforces the importance of early detection in managing this aggressive yet treatable disease. The results of this patient indicate that the collaborative efforts of the multidisciplinary team (MDT) are instrumental in the comprehensive management of IBC. This case is helpful in enhancing the comprehension of the diagnosis and treatment of IBC and guiding the clinical exploration of strengthening the individualized diagnosis and treatment strategies for breast cancer patients. In this study, the MDT thoroughly discussed the patient’s diagnosis, neoadjuvant chemotherapy regimen, surgical plan, and adjuvant treatment regimens. The postoperative pathological diagnosis achieved pCR, and no evident tumor recurrence or metastasis has been observed at present.

## 2. Case Presentation

We report a case of a 31‐year‐old female who had come to the breast department with a complaint of a lump in her left breast approximately four months ago. Initially, the lump was rigid with mild skin redness, but the patient did not receive treatment. Over the next 3 months, the lump was significantly enlarged and extended to the entire area of the left breast, and redness and swelling were observed. An ultrasonogram revealed a mixed left breast lump with enlarged axillary lymph nodes. She had no history of nipple discharge, fever, trauma, obesity, or family history of breast cancer but experienced weight loss of approximately 5 kg during the course of the disease.

**FIGURE 1:**
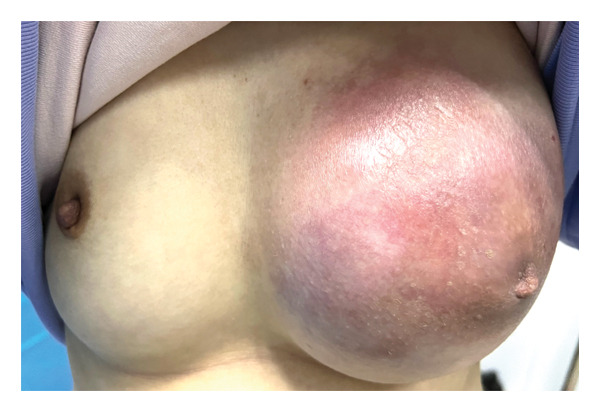
On the first visit of the patient: asymmetrical bilateral breasts, the lump almost occupied the whole left breast with skin redness and swelling.

On physical examination, the lump occupied most of the whole left breast. The following characteristics were observed: redness and swelling of the skin, unclear boundaries, irregular morphology, poor mobility, and tenderness. Palpation of the left axilla disclosed multiple nodes that were firm and enlarged, and exhibited matting and fixation. No lymphadenopathy was appreciated in the left supraclavicular area. The right breast and axilla were without palpable findings, and a complete systemic exam was otherwise normal (Figure 1).

**FIGURE 2:**
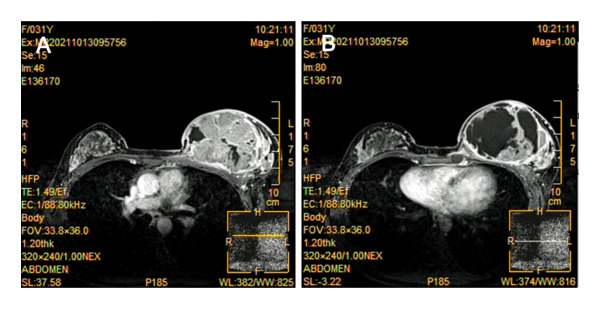
MRI demonstrated a significantly enlarged size of the left breast, demonstrating a breast mass and abnormal enhancement.

**FIGURE 3:**
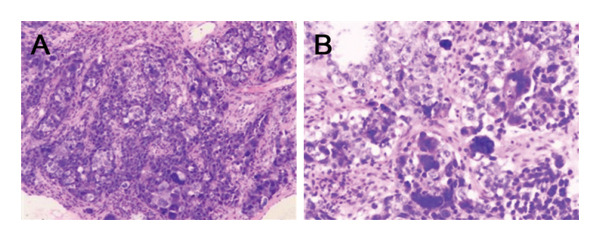
Core needle biopsy of the left breast mass. (A) H&E staining 200x. (B) H&E staining 400x.

**FIGURE 4:**
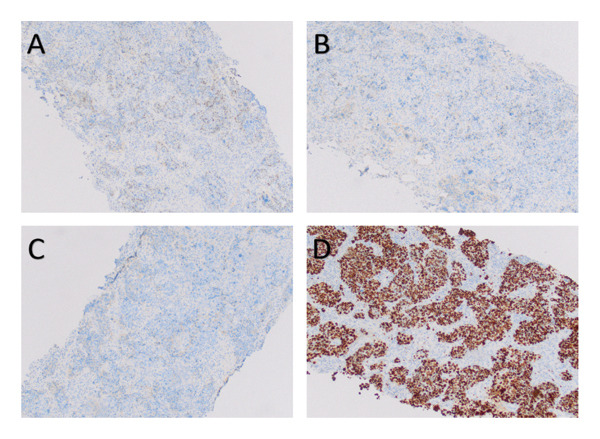
Immunohistochemistry of the left breast mass. (A) Estrogen receptor 100x. (B) Progesterone receptor 100x. (C) Human epidermal growth factor receptor 2 100x. (D) Ki‐67 100x.

**FIGURE 5:**
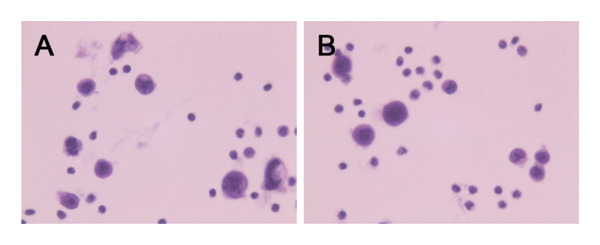
Core needle biopsy of left axillary and supraclavicular lymph nodes puncture. (A) Left axillary. (B) Supraclavicular (H&E staining 400x).

**FIGURE 6:**
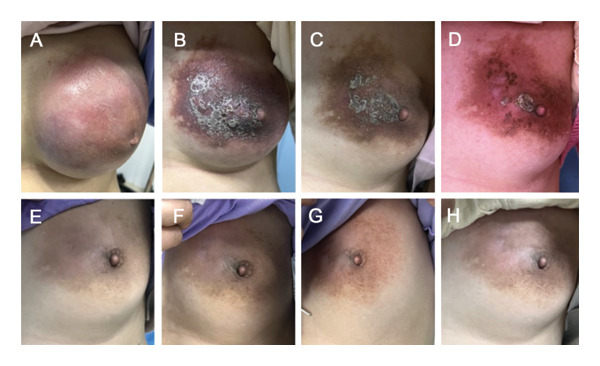
The appearance of the left breast mass changed during the neoadjuvant chemotherapy. (A–D) Changes during EC regimen. (E–H) Changes during the TP regimen.

**FIGURE 7:**
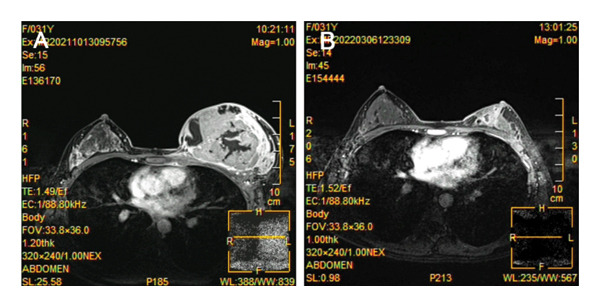
MRI contrast before and after neoadjuvant therapy. (A) Before neoadjuvant therapy. (B) After neoadjuvant therapy in 8 cycles.

An ultrasonogram revealed a 103 × 103 × 50 mm mixed lump with multilobate and clear boundaries in the left breast, and multiple enlarged lymph nodes were detected in the left axilla. The MRI of the left breast reveals the following: an enlarged capacity is observed, accompanied by notable uneven enhancement, diffuse skin thickening, and mild retraction of the nipple (Figure 2). Baseline staging evaluations (Sept. 2021), including abdominal ultrasound, chest CT, and bone scintigraphy, were negative for distant metastases. This patient refused to have a gene detection of BRCA1/2 and PET‐CT due to economic reasons. A core needle biopsy of the left breast mass was performed: postoperative pathology showed that the left breast mass was a poorly differentiated invasive carcinoma, dermal lymphatic tumor emboli were not definitively observed, and the histologic was Grade III (Figure 3). Immunohistochemistry (IHC): estrogen receptor (ER) (+1%–3%), progesterone receptor (PR) (−), human epidermal growth factor receptor 2 (Her‐2) (−), Ki‐67 (80%) (Figure 4). Following the puncture biopsy of the left axillary and the left supraclavicular lymph node, the pathology report revealed the presence of a significant number of carcinoma cells (Figure 5). According to the medical history and histopathological analysis, the tumor was diagnosed as left breast invasive carcinoma (low ER expression, PR negative, HER‐2 negative, stage cT4dN3cM0 IIIC). The neoadjuvant chemotherapy regimen was established based on the MDT discussion. Due to that situation, the recommended chemotherapy regimen by the MDT was the EC‐TP (epirubicin 90 mg/m^2^ plus cyclophosphamide 600 mg/m^2^ for four cycles followed by nab‐paclitaxel 260 mg/m^2^ plus carboplatin based on area under the curve of 5 for four cycles). As primary prophylaxis, a mecapegfilgrastim injection was used throughout neoadjuvant chemotherapy (Figure 6). During the chemotherapy treatment, laboratory tests revealed no signs of chemotherapy‐induced neutropenia (CIN) or neutropenic fever (FN) (Figure 7).

**FIGURE 8:**
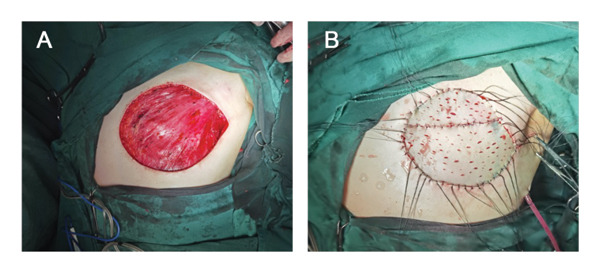
An operative photograph of the patient. (A) Intraoperative skin defect and (B) intraoperative split‐thickness skin graft.

**FIGURE 9:**
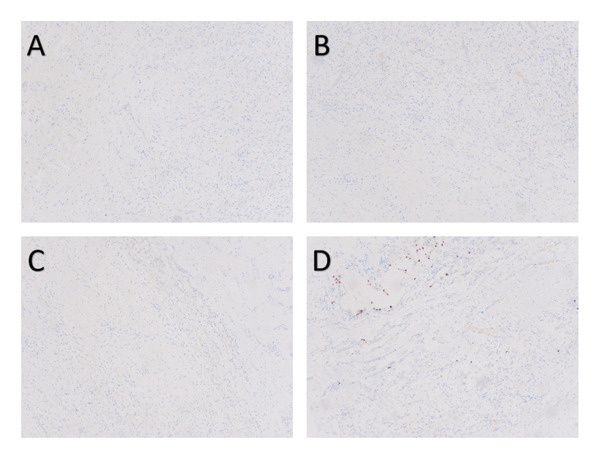
Immunohistochemistry of the left breast mass postoperatively. (A) Estrogen receptor 100x. (B) Progesterone receptor 100x. (C) Human epidermal growth factor receptor 2 100x. (D) Ki‐67 100x.

The patient underwent modified radical resection for left breast cancer, which encompassed the dissection of the axillary Level I, Level II, and subclavian lymph nodes, accompanied by split‐thickness skin graft (Figure 8). The postoperative pathological results showed (1) a tumor measuring approximately 4.5 ∗ 3 cm in size was observed. (2) Microscopically, the medical history and histopathology revealed cellular response to chemotherapy (Miller‐Payne Grade 4). IHC: ER (−), PR (−), HER‐2 (−), CK5/6 (−), (Miller‐Payne Grade 4) (Figure 9). The ipsilateral axillary examination revealed that none of the 39 harvested lymph nodes contained metastases, and three of the lymph nodes exhibited a substantial presence of foam cells.

During the postoperative MDT, the topic of further systemic treatment was discussed. Based on MDT discussion and additional pathological findings provided by the patient, we revised the postoperative diagnosis: ypTisN0M0, with low ER expression, PR negative, and HER‐2 negative (Miller‐Payne Grade: 5). According to the recommendations of the MDT, the following treatment plan was formulated: firstly, the direction of radiotherapy for the chest wall and the axillary and supraclavicular drainage areas was determined; secondly, the experts recommended intensive adjuvant chemotherapy with capecitabine. Furthermore, there is no sufficient evidence to support the implementation of adjuvant endocrine therapy in cases of low ER expression. However, a retrospective study revealed that patients exhibiting low ER expression demonstrated a reduced risk of recurrence following 2 to 3 years of endocrine therapy, yet the risk did not exhibit a significant disparity when compared to the risk observed following 5 years of endocrine therapy [6]. Consequently, the consensus among experts on MDT is that short‐term endocrine therapy should be administered to the patient.

The patient received adjuvant radiotherapy, endocrine therapy, and intensive therapy with capecitabine in April 2022. Up to January 2026, there was no obvious propensity for tumor recurrence in the patient’s follow‐up.

## 3. Discussion

IBC is the most aggressive subtype of LABC, accounting for between 1% and 5% of newly diagnosed cases of breast cancer. Compared to noninflammatory LABC, IBC is characterized by its rapid disease progression, poor response to treatment, and a high risk of recurrence and metastasis in the short term [7, 8]. Consequently, there are significant differences in multimodal treatment (including systemic chemotherapy, surgery, and radiotherapy) and recurrence risk. A crucial challenge in the diagnosis of breast diseases in young women is the distinction between mastitis, nonlactating mastitis, and nonspecial types of breast cancer. After a thorough review of the patient’s medical history, comprehensive physical examination, adjuvant examination, and pathological diagnosis, it is imperative to clarify the diagnosis by the Clinical Practice Guidelines for the Diagnosis and Treatment of IBC.

Similar to non‐IBC, confirming molecular subtype is a significant method for comprehensively treating IBC. In this case, the patient showed low ER expression and was PR negative and HER‐2 negative and received EC‐TP as a neoadjuvant chemotherapy regimen made through MDT discussion. According to the extant research, the implementation of dose‐intensive dosing regimens has been demonstrated to effectively prolong disease‐free survival and/or overall survival in patients diagnosed with breast cancer that has spread to the lymph nodes or aggressive lymphoma [9]. A meta‐analysis revealed that the prophylactic use of G‐CSF significantly led to a substantial decrease in the incidence of infection and the risk of neutropenia [10]. Some randomized trials of 17 patients with solid tumors and 3493 patients with lymphoma found that G‐CSF, when used as primary prophylaxis, reduced the risk of FN by 46%, with Pegylated Recombinant Human Granulocyte Colony‐Stimulating Factor (PEG‐rhG‐CSF) showing a 92.3% reduction in risk. Furthermore, as a primary prophylactic measure, G‐CSF demonstrated a 44.8% and 40.1% reduction in the risk of infection‐related death and early mortality, respectively, during chemotherapy [11]. In this report, the follow‐up laboratory test data of the patient did not have CIN or FN during chemotherapy, showing the effectiveness of the implemented preventive strategy [12].

In previous retrospective studies, the pCR rate for those who had complete neoadjuvant chemotherapy for IBC ranged from 17% to 39%. Currently, especially for triple‐negative IBC, the standard therapy remains dose‐intensive chemotherapy options [13]. Therefore, the addition of platinum drugs into neoadjuvant chemotherapy regimens has been demonstrated to enhance the pCR rate in patients diagnosed with triple‐negative, non‐IBC [14]. However, there is no relevant research to support this effect in IBC. In addition, platinum‐based treatment regimens in patients with triple‐negative breast cancer (TNBC) with BRCA mutations exhibited superior clinical outcomes in comparison to those with BRCA wildtype [15]. This observation suggests a potential sensitivity of patients with BRCA mutations to platinum‐based chemotherapy. Furthermore, the KEYNOTE‐522 study demonstrated that the pembrolizumab combined with the neoadjuvant EC‐TP regimen led to a substantial enhancement in the pCR rate among patients with TNBC, including those with triple‐negative IBC [16]. Consequently, immunotherapy combined with chemotherapy in patients diagnosed with triple‐negative IBC has the potential to evolve into a prevailing therapeutic modality in the future.

For patients with IBC who achieve pCR after neoadjuvant systemic therapy, the optimization of local regional treatment remains a critical research area. Although pCR is associated with a better prognosis than residual lesions, IBC patients still face a persistent risk of local regional recurrence (LRR), which may not be fully mitigated by systemic therapy alone [17]. Recent studies have demonstrated that all observed local recurrences in patients receiving triple therapy occurred in those who did not achieve pCR, highlighting the superior local control outcomes achievable with aggressive local therapy in pCR patients [18]. Per current guidelines, the standard treatment paradigm for IBC comprises a modified radical mastectomy (with resection of all clinically involved skin) followed by postoperative radiotherapy. This approach is essential for eliminating potential microscopic residual disease within dermal lymphatics [19, 20]. Although de‐escalation strategies such as sentinel lymph node biopsy or breast‐conserving surgery have been explored, the current body of evidence does not support their routine application in IBC, even in the setting of pCR [20, 21]. Therefore, in the absence of prospective data confirming the safety of de‐escalated approaches in this distinct patient population, complete trimodality therapy remains the established standard of care for all patients with nonmetastatic IBC, regardless of treatment response [17, 19, 22].

Due to the lack of relevant studies, the use of endocrine therapy for patients with low ER expression remains controversial. According to the 2020 ASCO/CAP Guidelines, there is currently no clear evidence supporting the efficacy of endocrine therapy for patients exhibiting low ER expression. However, the guidelines acknowledge the existence of limited data that suggests patients with low levels of ER positivity may still derive benefits from endocrine therapy and exhibit improved outcomes compared to those with ER‐negative tumors. In this case, the patient could benefit significantly from endocrine therapy.

In conclusion, this case contributes to the limited literature on IBC patients achieving pCR, reinforcing the importance of continued aggressive locoregional therapy even in good responders, while also highlighting the EC‐TP regimen as a viable neoadjuvant option in this challenging clinical entity.

## Funding

This study was supported by the Health Research Program of Anhui, AHWJ2023BAc20034.

## Consent

The consent was obtained from the patient.

## Conflicts of Interest

The authors declare no conflicts of interest.

## Data Availability

The data that support the findings of this study are available from the corresponding author upon reasonable request.
